# AI-based modeling of treatment decisions in benign prostatic hyperplasia: a transformer-based comparative study

**DOI:** 10.1186/s12911-026-03438-9

**Published:** 2026-03-17

**Authors:** Mohammad Alshraideh, Bahaaldeen Alshraideh, Abedalrahman Alshraideh, Bayan Alfayoumi, HebaAlshraideh

**Affiliations:** 1 Artificial Intelligence Department, The University of Jordan, Amman, Jordan; 2 The Information Technology College, Lusail University, Lusail, Qatar; 3 Department of Special Surgery, The University of Jordan, Amman, Jordan; 4 Internal Medicine, East Midlands Deanery, NHS, England, UK; 5 Medicine School, The University of Jordan, Amman, Jordan

**Keywords:** Clinical decision support systems, Prostate disease stratification, Treatment pathway optimization, Transformer-based deep learning, Outcome-oriented risk prediction, Precision urology

## Abstract

Benign Prostatic Hyperplasia (BPH) is a common condition among aging men that often causes significant urinary symptoms, impacting their quality of life. This study employs advanced LLMs and deep learning models to predict whether BPH patients were managed with TURP or continued medical therapy using historical clinical data. We utilized a dataset of 883 patient cases from Jordan University Hospital (JUH), comprising 15 clinical attributes, including PSA levels, prostate size, and treatment history. Five models were tested: GEMMA, GPT, and three deep learning models—Recurrent Neural Network (RNN), Convolutional Neural Network (CNN), and Long Short-Term Memory (LSTM). The GEMMA model achieved the highest performance, with an accuracy of 92% and an ROC AUC score of 0.94. The GPT model was a close second with 91% accuracy, demonstrating its robustness in handling multimodal data. Deep learning models showed promising results, with LSTM outperforming CNN and RNN because of its ability to capture sequential dependencies. The findings emphasize the importance of choosing relevant features, like PSA levels and prostate size, for better prediction accuracy, which significantly impacts the decision to either keep medication or move to surgery.

**Supplementary Information:**

The online version contains supplementary material available at 10.1186/s12911-026-03438-9.

## Introduction

Benign Prostatic Hyperplasia (BPH) is a common condition among aging men, leading to an enlarged prostate gland [1], which can cause urinary symptoms and greatly affect quality of life. As the global population ages, the prevalence of BPH is expected to increase, making its diagnosis and treatment a crucial concern in healthcare [2]. Deciding on the appropriate treatment—whether medication or surgical procedures, such as Transurethral Resection of the Prostate (TURP)—requires careful evaluation of various factors [3], including patient symptoms, medical history, and clinical test results. This process often involves weighing the benefits and risks of different options to achieve the best outcome for the patient [4].

In clinical practice, BPH treatment decisions are typically based on a combination of patient-reported symptoms, clinical examinations, and laboratory test results, including prostate-specific antigen (PSA) levels, prostate size [5], and peak urinary flow rates. However, this process is subjective and can be influenced by a physician’s experience, patient preferences, and disease complexity [6]. Given the complexities involved in assessing and selecting the most effective treatment for BPH patients [7], there is an excellent opportunity to leverage advanced machine learning and artificial intelligence (AI) models to improve decision-making accuracy, predict patient responses, and recommend the most appropriate treatment options [8].

Recent advancements in AI, including deep learning techniques such as Convolutional Neural Networks (CNN), Recurrent Neural Networks (RNN), Long Short-Term Memory (LSTM) networks, and gradient boosting models (GBT), have shown considerable promise in various medical domains, particularly in predictive modeling and classification tasks [9]. These methods enable the processing of vast amounts of clinical data and the identification of patterns that may not be immediately apparent through traditional statistical approaches. By integrating clinical features and outcomes, AI models may assist clinicians by providing data-driven risk stratification insights that complement, rather than replace, physician judgment.

While transformer architectures were originally developed for natural language processing tasks, recent advances have demonstrated their applicability to structured tabular data. Transformers are particularly well-suited for modeling complex nonlinear relationships and higher-order feature interactions through self-attention mechanisms. In clinical datasets, where multiple interdependent variables (e.g., PSA levels, prostate size, renal function, urinary retention frequency) jointly influence decision-making, attention-based architectures may capture interactions that traditional linear approaches do not explicitly model. Emerging studies have reported competitive or superior performance of transformer-based models in tabular medical prediction tasks, supporting their exploration in this context.

This study is significant because it fills a critical gap in BPH treatment decision-making by using advanced machine learning and deep learning techniques to predict whether a patient should undergo TURP or continue medical therapy. Considering the high prevalence of BPH and its substantial impact on patients’ lives, optimizing the treatment decision process is extremely important. Accurate predictions of treatment success and potential complications can enhance patient outcomes, reduce healthcare costs, and inform more personalized treatment plans. Furthermore, providing physicians with AI-driven tools that deliver data-backed insights can help standardize care, minimize treatment variation, and reduce the risk of suboptimal decision-making.

By examining a dataset from Jordan University Hospital (JUH) in Amman, Jordan, which includes key clinical features such as PSA levels, prostate size, and uroflowmetry measures, the study aims to determine the most relevant factors influencing the decision between medical therapy and transurethral resection of the prostate (TURP). The findings from this research could significantly impact clinical guidelines for the management of benign prostatic hyperplasia (BPH), leading to a more evidence-based approach to patient care. Furthermore, advanced machine learning models, such as GEMMA, GPT, CNN, RNN, and LSTM, can enhance predictive accuracy and efficiency in decision support systems, ultimately benefiting patients and healthcare providers.

As AI revolutionizes healthcare, this study is a step forward in integrating cutting-edge technology into clinical practice. The implications extend beyond BPH treatment, as the methodologies developed here can be adapted for other medical conditions that require complex, multifactorial decision-making.

## Background and related works

### Background

Benign Prostatic Hyperplasia (BPH) is a common and progressive condition in aging men that often causes an enlarged prostate gland, leading to urinary symptoms such as increased frequency, urgency, and difficulty urinating. The prevalence of BPH increases with age, and by age 60, about 50% of men will experience symptoms. By age 80, this can rise to as much as 90%. The condition’s underlying mechanism involves hormonal changes, especially the effects of dihydrotestosterone (DHT), a potent androgen that stimulates prostate growth. Although BPH is not cancerous, it can severely affect a patient’s quality of life. If left untreated, it may lead to serious complications like urinary retention, bladder stones, and kidney failure [10].

BPH is often diagnosed based on patient-reported symptoms, clinical evaluations, and laboratory tests. The most commonly used diagnostic tool is the International Prostate Symptom Score (IPSS), which measures urinary symptom severity. In addition to symptom scores, clinical assessments such as digital rectal exams (DRE) and ultrasonography are used to determine prostate size and rule out malignancy. Key laboratory tests, such as serum prostate-specific antigen (PSA) levels, are crucial for diagnosis. Elevated PSA levels are frequently associated with BPH, but they can also indicate prostate cancer, so distinguishing between these conditions is necessary [11]. Another important diagnostic test for BPH is uroflowmetry, which measures urine flow rate and helps assess the degree of urinary obstruction caused by an enlarged prostate. Measuring post-void residual volume is also helpful to determine how much urine remains in the bladder after urination, providing additional insight into the severity of the condition [12].

Several factors, including symptom severity, complications, and patient preferences, influence treatment decisions for benign prostatic hyperplasia (BPH). The main treatment options are medical therapy, minimally invasive procedures, and surgical intervention. Medical management usually involves alpha-blockers, which relax the smooth muscles of the prostate and bladder neck, improving urine flow. Another common approach is the use of 5-alpha reductase inhibitors (such as finasteride), which decrease the size of the prostate by blocking the conversion of testosterone to DHT. Dual therapy, combining both alpha-blockers and 5-alpha reductase inhibitors, is often used for patients with moderate to severe symptoms [13]. While medication works well for many patients, those who do not respond adequately or develop complications may need surgical intervention.

Transurethral resection of the prostate (TURP) is the gold-standard surgical procedure for BPH and involves the removal of excess prostate tissue using a resectoscope inserted through the urethra. Although TURP is highly effective in relieving symptoms and preventing complications, it has risks. These include bleeding, infection, and erectile dysfunction. Given the potential for complications and the procedure’s invasiveness, the decision to proceed with TURP should be carefully considered [14].

In recent years, the integration of artificial intelligence (AI) and machine learning (ML) techniques has significantly advanced medical decision-making, including the diagnosis and treatment of benign prostatic hyperplasia (BPH). AI models, such as support vector machines (SVMs), decision trees, and neural networks, have been used to analyze clinical data, predict treatment outcomes, and assist healthcare professionals in making more informed, personalized treatment decisions. These models can handle large amounts of diverse data, including patient demographics, clinical findings, and lab results, to assist in clinical decision-making [15]. The development of AI models for BPH treatment aims to optimize resource allocation and enhance patient outcomes by predicting which patients are most likely to benefit from specific treatments, such as medical therapy or transurethral resection of the prostate (TURP).

Recent studies have shown that machine learning algorithms can accurately predict BPH progression and treatment responses, including Random Forests, Gradient Boosting Machines (GBMs), and Artificial Neural Networks (ANNs). For example, a study by Singh et al. (2021) demonstrated that Random Forest models could predict the likelihood of a patient progressing to TURP with an accuracy of over 85% [16]. Similarly, another study by Lee et al. (2022) applied a gradient-boosting model to BPH clinical data. It achieved promising results in predicting treatment efficacy, providing clinicians with valuable insights for tailoring treatment strategies [17].

Furthermore, deep learning techniques such as Convolutional Neural Networks (CNNs), Recurrent Neural Networks (RNNs), and Long Short-Term Memory (LSTMs) have been studied in the medical field for their ability to process sequential data and recognize complex patterns in time-series data. CNNs, primarily used for image recognition tasks, have been adapted to analyze medical images, including prostate ultrasound images, to aid in diagnosis and treatment planning. RNNs and LSTMs, however, are particularly well-suited to time-series data, such as monitoring patient progress over time or analyzing treatment responses [18]. Incorporating these deep learning models into BPH management could significantly enhance predictive accuracy, enabling clinicians to identify patients who may benefit most from surgery versus those who can continue with medical therapy.

Integrating AI into BPH management can help reduce healthcare costs by optimizing resource utilization and preventing unnecessary procedures. For example, AI can identify patients who are unlikely to benefit from surgery, thereby avoiding costly procedures that also carry risks. Furthermore, AI-powered decision support systems could help standardize treatment protocols and reduce disparities across different healthcare settings, ensuring patients receive the most appropriate treatment based on their individual characteristics [19].

Despite the potential benefits, implementing AI in clinical decision-making for benign prostatic hyperplasia (BPH)presents several challenges. Data quality, model interpretability, and the need for large, diverse datasets remain important considerations [20]. Additionally, adopting AI in clinical practice requires careful model validation to ensure it is accurate, reliable, and safe for use in real-world settings. Furthermore, ethical concerns regarding patient privacy, data security, and the role of AI in healthcare decision-making must be addressed to ensure that these technologies are used responsibly and equitably [21].

The decision-making process for BPH treatment is complex and influenced by multiple factors, including patient age, symptom severity, prostate volume, PSA levels, comorbidities, and patient preferences. At the same time, guidelines exist to aid treatment decisions, yet variability in clinical practice persists, with treatment choices often reflecting individual physician experience and patient expectations [22].

Accurately predicting treatment outcomes and potential complications is crucial in guiding clinical decisions. Larger prostate volumes and elevated PSA levels often indicate the need for surgical intervention, while patients with smaller prostate volumes and stable PSA levels may be managed successfully with medication. However, predicting the progression of BPH and the response to treatment remains challenging, necessitating the development of more precise tools for patient assessment [23].

In BPH management, machine learning models have been developed to predict the likelihood of acute urinary retention, assess surgical complication risk [24, 36], and evaluate the response to medical therapy. Studies have shown that machine learning algorithms can accurately predict the need for surgical intervention in patients with benign prostatic hyperplasia (BPH) based on clinical and laboratory data, thereby helping clinicians make informed treatment decisions.

By integrating machine learning into clinical practice, healthcare providers can leverage predictive models to significantly enhance patient care, reduce healthcare costs, and, most importantly, improve patient outcomes. Developing a decision-support tool incorporating machine learning could provide valuable insights into treatment planning, optimize resource utilization, and facilitate shared decision-making between clinicians and patients [25]. This potential to improve patient outcomes is a source of hope and positivity for the healthcare community.

### Related works

There is growing interest in harnessing machine learning to revolutionize the diagnosis and management of Benign Prostatic Hyperplasia (BPH). Traditional clinical approaches often rely on subjective assessments and generalized treatment protocols, which may not adequately account for the unique characteristics and needs of individual patients [26]. To address this limitation, researchers have increasingly turned to machine learning to develop more personalized and precise treatment strategies for BPH, sparking a wave of optimism and inspiration in the healthcare community. Recent advancements in machine learning have significantly impacted the management of benign prostatic hyperplasia (BPH), providing new tools for enhancing diagnostic accuracy and treatment outcomes. One notable study by Kim et al. [27] used Random Forests and Support Vector Machines (SVMs) to predict acute urinary retention (AUR) in patients with BPH, achieving 93% accuracy, underscoring the potential of these algorithms to stratify patients by risk profile. Similarly, Chen et al. [28] employed neural networks to support decision-making for surgical interventions, with their model predicting surgical outcomes and complications, achieving an AUC-ROC of 0.91. This demonstrates the utility of machine learning in optimizing surgical planning. In imaging, Nguyen et al. [29] applied Convolutional Neural Networks (CNNs) to MRI data, achieving a Dice coefficient of 0.92 for prostate segmentation, thereby enhancing diagnostic precision. Li et al. [30] extended this approach by integrating CNNs with Recurrent Neural Networks (RNNs) to analyze time-series data from uroflowmetry tests. This integration achieved 89% accuracy in predicting urinary flow patterns, facilitating early detection of abnormal flow dynamics. The application of Natural Language Processing (NLP) has also been explored; Sato et al. [31] used transformer-based models to extract and analyze data from electronic health records (EHRs), achieving 85% precision in identifying key disease indicators. Tan et al. [32] further used sentiment analysis and topic modeling in NLP to correlate patient-reported outcomes with treatment types, providing insights into patient satisfaction and treatment efficacy.

Additionally, machine learning has been applied to surgical planning. The authors [33, 37] developed a Random Forest model that predicted postoperative complications with 90% accuracy, assisting clinicians in preoperative planning. Miller et al. [34] explored the use of Reinforcement Learning (RL) to recommend individualized surgical strategies based on patient-specific factors, potentially improving surgical success rates. The integration of wearable technology has also been examined by Lee et al. [35], who combined real-time wearable data with machine learning to monitor BPH symptoms, thereby enhancing patient engagement and adherence to treatment plans. Collectively, these studies illustrate the transformative impact of machine learning on BPH management, from predicting treatment outcomes and optimizing surgical interventions to improving diagnostic accuracy and personalizing patient care. Future research should continue to refine these models and integrate them into clinical practice to enhance patient outcomes further and streamline BPH management.

**Table 1:** A comparison of recent studies on the application of machine learning in Benign Prostatic Hyperplasia (BPH) management

Study	Objective	Machine Learning Algorithm	Dataset Size	Key Features	Key Results	Accuracy / Metric
Kim et al. [27]	Predict acute urinary retention (AUR)	Random Forest, Support Vector Machines (SVM)	1,000 patients	PSA levels, prostate volume, urinary symptoms	Predicts AUR risk	93% accuracy
Chen et al. [28]	Support surgical intervention decisions	Neural Networks	Data on TURP patients	Patient demographics, preoperative findings	Predicts surgical outcomes and complications	ROC AUC of 0.91
Nguyen et al. [29]	Analyze MRI data for prostate segmentation	Convolutional Neural Networks (CNNs)	MRI images	Prostate volume, structural abnormalities	High accuracy in prostate segmentation	A dice coefficient of 0.92
Li et al. [30]	Predict urinary flow patterns using time-series data	CNNs with Recurrent Neural Networks (RNNs)	Uroflowmetry data	Urinary flow rate patterns, time-series data	Predicts abnormal flow dynamics	89% accuracy
Sato et al. [31]	Extract and analyze data from EHRs for BPH patients	Transformer-based NLP models	EHR data	Clinical notes, disease indicators	Extract relevant information from clinical notes	Precision of 85%
Tan et al. [32]	Analyze patient-reported outcomes for treatment efficacy	NLP with sentiment analysis and topic modeling	Patient feedback	Treatment type, symptom improvement, and patient satisfaction	Correlates outcomes with treatment and satisfaction	N/A (focus on correlation)
Brown et al. [33]	Predict surgical outcomes and complications	Random Forest	800 patients	Prostate size, preoperative symptoms, and comorbidities	Predicts postoperative complications	90% accuracy
Miller et al. [34]	Recommend individualized surgical strategies	Reinforcement Learning (RL)	Surgical data	Patient-specific factors, historical outcomes	Provides tailored surgical recommendations	N/A (focus on recommendations)
Lee et al. [35]	Monitor BPH symptoms using wearable technology	Machine Learning with wearable data	Wearable data	Urinary patterns, fluid intake, and physical activity	Enhances symptom monitoring and patient engagement	N/A (focus on monitoring

Table 1 compares the objectives, machine learning algorithms, dataset sizes, key features, and results across various studies on BPH management. It provides a clear overview of how different approaches and technologies are applied to enhance diagnosis, treatment, and patient care.

While recent studies have emphasized the role of machine learning in predicting outcomes for BPH patients, few have explicitly connected these models to established clinical practice guidelines. According to the European Association of Urology (EAU, 2024) [38] and the American Urological Association (AUA, 2023) [39], key factors for surgical intervention include prostate volume, severity of lower urinary tract symptoms (LUTS), recurrent urinary retention, and complications such as bladder stones or renal impairment. These factors overlap significantly with the features prioritized by our models, especially PSA levels, prostate size, and urinary retention. By showing agreement between AI-predicted features and guideline-recommended decision criteria, this study reinforces the case for incorporating AI-driven tools into existing clinical workflows. Such alignment ensures that predictive models are not only technically sound but also clinically relevant, increasing their likelihood of acceptance by practicing urologists.

## Methodology

This study uses machine learning algorithms to predict the most effective treatment options for patients with benign prostatic hyperplasia (BPH). The research leverages a comprehensive dataset from the Jordan University Hospital (JUH) in Amman, Jordan. This methodology outlines a systematic approach to data preprocessing, selecting appropriate machine learning models, and evaluating their performance.

### Study population and eligibility criteria

We retrospectively identified consecutive male patients evaluated for symptomatic benign prostatic hyperplasia (BPH) at a tertiary referral center between January 2017 and December 2024. Inclusion criteria comprised age ≥ 40 years, clinical diagnosis of non-neurogenic male lower urinary tract symptoms (LUTS) attributed to BPH, documented prostate volume assessment, PSA measurement, and baseline uroflowmetry parameters. Patients were excluded if they had prior prostate surgery, known or newly diagnosed prostate cancer at baseline, neurogenic bladder dysfunction, urethral stricture disease, active urinary tract infection at evaluation, or incomplete key predictor variables.

Eligible patients were identified through systematic electronic medical record screening using standardized diagnostic and procedural codes. All consecutive cases meeting the inclusion criteria during the study period were included to reduce selection bias and improve cohort representativeness.

This cohort reflects real-world tertiary BPH management during the contemporary guideline era (2017–2024).

### Dataset collection & description

#### Patient selection and eligibility criteria

This retrospective study included consecutive adult male patients diagnosed with benign prostatic hyperplasia (BPH) who were managed at Jordan University Hospital between January 2018 and December 2023. Patients were identified from electronic medical records using diagnostic codes for non-neurogenic male lower urinary tract symptoms (LUTS) secondary to BPH.

**Inclusion criteria** were: (1) age ≥ 45 years, (2) documented diagnosis of BPH confirmed by clinical assessment and ultrasonography, (3) availability of complete baseline laboratory and imaging data relevant to the study variables, and (4) documented treatment decision (continued medical therapy or TURP).

**Exclusion criteria** included: (1) confirmed or suspected prostate malignancy, (2) prior prostate surgery before the indexed treatment decision, (3) neurogenic bladder disorders, (4) severe renal failure requiring dialysis, and (5) incomplete key clinical variables after imputation feasibility assessment.

Consecutive case identification was applied to minimize selection bias. All eligible cases during the study period were screened, and no sampling or random selection was performed. The final dataset comprised 883 patients who met the eligibility criteria.

The dataset used in this study consists of 883 cases of BPH patients treated at JUH. The dataset is divided into two groups: 477 cases in which patients are treated with medication and 406 cases in which patients undergo Transurethral Resection of the Prostate (TURP). The data captures clinical, demographic, and laboratory features relevant to BPH diagnosis and treatment decisions. The 15 features included in the dataset are as follows:

1. **Age**: The Patient’s age at the time of consultation.
2. **Number of Months on Dual Therapy**: Duration (in months): The Patient has been on a combination therapy of alpha-blockers and finasteride.
3. **PSA Levels**: Total prostate-specific antigen levels in the blood.
4. **Free PSA Levels**: Unbound PSA levels in the blood provide additional diagnostic information.
5. **PSA Ratio**: The free PSA-to-total PSA ratio indicates the potential risk of prostate cancer.
6. **Creatinine Levels**: Blood creatinine levels indicate kidney function and overall metabolic health.
7. **HbA1c**: Glycated hemoglobin levels, reflecting long-term blood sugar control, particularly in diabetic patients.
8. **Microscopic Hematuria**: Blood cells were detected in the urine during the last urinalysis.
9. **Recurrent Urinary Tract Infections (UTIs)**: The number of urinary tract infections experienced by the Patient based on urine culture results.
10. **Urinary Retention**: The number of urinary retention episodes indicates potential bladder dysfunction.
11. **History of Bladder Stones**: Past occurrences of bladder stones, which may complicate BPH.
12. **Previous History of Cardiac Catheterization**: Include any prior cardiac catheterization procedures relevant to assessing overall patient health.
13. **Peak Flow Rates (Qmax) on Uroflowmetry**: The Maximum urinary flow rate measured during Uroflowmetry, indicating the severity of urinary obstruction.
14. **Post-Void Residual**: The volume of urine remaining in the bladder after urination, measured through ultrasound
15. **Prostate Size on Ultrasound**: Prostate volume is assessed via ultrasound, providing insight into the severity of prostate enlargement.

The target variable in this study reflects the historical treatment decision recorded in the electronic medical record (medical therapy vs. TURP). The model, therefore, learns patterns associated with prior physician decision-making rather than independently determining clinical necessity. It is important to emphasize that the system is intended as a decision-support aid and does not replace clinician judgment or prescribe treatment. Clinical appropriateness must always be evaluated within the broader patient context.

### Data preprocessing

#### Data cleaning

A structured preprocessing pipeline was implemented to ensure data integrity, minimize bias, and prevent information leakage. Missing values were addressed using a predefined imputation protocol tailored to variable type and distribution. For continuous variables (e.g., PSA, Free PSA, creatinine, HbA1c, and post-void residual volume), median imputation was applied for skewed distributions, while mean imputation was used for approximately normally distributed variables. Categorical variables (e.g., history of bladder stones and prior cardiac catheterization) were imputed using mode imputation. In cases where multivariate dependency was suspected, K-Nearest Neighbor (KNN) imputation was performed within the training folds to preserve cross-validation integrity.

Outlier detection was conducted using complementary statistical approaches. Z-score analysis was applied to continuous variables such as age, PSA levels, and prostate size, with values exceeding ± 3 standard deviations flagged for review. The Interquartile Range (IQR) method was used for skewed or count-based variables (e.g., recurrent urinary tract infections and post-void residual), identifying observations outside the 1.5 × IQR limits. Outliers were assessed clinically before correction or retention to avoid removing clinically plausible extreme values.

To ensure measurement consistency, laboratory values were standardized to a uniform set of units (e.g., PSA converted to a single reporting unit). Continuous variables were normalized using Min–Max scaling to the range [0,1]. Importantly, scaling parameters were fitted exclusively on training data within each cross-validation fold and subsequently applied to validation and test partitions, thereby preventing data leakage.

Categorical variables were encoded using binary encoding (Yes/No → 0/1) or ordinal mapping when clinically justified. For transformer-based models, structured tabular features were serialized into standardized textual templates to leverage pretrained language representations. Tokenization was performed using byte-pair encoding (BPE), ensuring consistent feature ordering and representation across all samples.

This preprocessing strategy ensured methodological rigor, preserved cross-validation validity, and maintained compatibility across both classical machine learning and transformer-based architectures.

#### Feature selection

In the feature selection phase, we employed a blend of statistical methods and domain expertise to identify the most relevant features for the model. Correlation analysis was performed using Pearson’s correlation coefficient for linear relationships and Spearman’s rank correlation for non-linear relationships and ordinal variables. This helped identify potential multicollinearity among features such as PSA levels, Free PSA levels, and the PSA ratio, which share closely related clinical implications. Mutual information scores were computed to determine the dependency between features and the target variable. This analysis highlighted features such as prostate size on ultrasound and peak flow rates (Qmax) as significantly informative for predicting treatment decisions, including the necessity of transurethral resection of the prostate (TURP). We further employed Recursive Feature Elimination (RFE) with models such as Random Forests and Decision Trees to rank features by predictive power, thereby validating the importance of age, creatinine levels, and post-void residual volumes. Domain experts, particularly urologists, were consulted to align feature selection with clinical relevance. Emphasized features included a history of bladder stones and prior cardiac catheterization, based on medical insights, to ensure that the selected features were both statistically and clinically significant.

To prevent data leakage, all feature selection procedures (correlation analysis, mutual information, and recursive feature elimination) were conducted exclusively within each training fold during cross-validation. No information from the validation or test sets was used during feature selection or model tuning.

#### Data splitting

The dataset was methodically divided to ensure the practicality of model training, validation, and testing. Initially, a train-test split was implemented, dividing the dataset into a training set (70%), a validation set (15%), and a testing set (15%). Stratified sampling was used to maintain the distribution of the target classes (medication and TURP) across all subsets. Stratification ensures that each subset accurately represents the overall dataset, thereby preventing bias and enhancing the model’s reliability and accuracy. We employed 5-fold cross-validation during model training to evaluate performance across multiple data partitions, reducing overfitting and providing a robust estimate of the model’s generalization capabilities. This method enables each data point to participate in both training and validation, providing a comprehensive performance assessment. For hyperparameter tuning, hold-out validation was employed, reserving a validation set to fine-tune model parameters without affecting the training data. Random shuffling was applied before splitting to distribute data points evenly and avoid any sequential bias that could affect model learning. Although the dataset was nearly balanced (477 medication vs. 406 TURP cases), balancing techniques such as oversampling or undersampling were deemed unnecessary, as models like Random Forests handle class imbalance through built-in weighting mechanisms. These careful splitting and validation strategies ensure that the model trained on this dataset is robust, accurate, and capable of making reliable treatment decisions for patients with benign prostatic hyperplasia (BPH). Importantly, preprocessing steps—including normalization, imputation, and feature scaling—were fit only to the training data within each fold and subsequently applied to the validation and test partitions. This ensured strict separation between training and evaluation data and eliminated potential leakage.

### Model evaluation

Evaluating the models is crucial for determining their effectiveness in predicting whether patients need Transurethral Resection of the Prostate (TURP) or should continue with medication-based treatment. Several advanced machine learning and deep learning algorithms were employed to assess their predictive accuracy, precision, recall, and F1 score. Specifically, the Gradient Boosting-based GEMMA and GPT-based large language models were selected due to their ability to handle complex interactions within the dataset, including patient-specific features such as PSA levels, creatinine, recurrent urinary retention, and prostate size. Deep learning approaches, including CNNs, RNNs, and LSTMs, complement these models well, as they are well-suited for analyzing structured and sequential data. In addition to advanced transformer and deep learning architectures, a classical Logistic Regression (LR) model was implemented as a statistical baseline comparator. Logistic regression was selected due to its widespread use in clinical risk prediction modeling and its interpretability in tabular data contexts.

The evaluation metrics included accuracy to measure overall correctness, ROC AUC scores to assess the models’ ability to distinguish between TURP and medication treatment cases, precision to minimize false positives, and F1-score to ensure a balanced tradeoff between precision and recall. GEMMA and GPT models demonstrated superior performance by capturing complex relationships among the 15 attributes, achieving accuracy rates exceeding 90%. Among the deep learning models, LSTM performed best due to its capability to handle sequential patient history data, followed by CNN and RNN. The results were validated using a stratified k-fold cross-validation approach to ensure robustness and minimize overfitting. This comprehensive evaluation identified the most suitable model to support clinical decision-making in patients with benign prostatic hyperplasia (BPH), aligning with the study’s objective of enhancing physician recommendations with AI-driven insights.

#### Accuracy

Accuracy measures the proportion of correctly classified samples among all samples. It is a general indicator of model performance but may not be fully informative in cases of class imbalance [26].

1 $$\mathrm{A}\mathrm{c}\mathrm{c}\mathrm{u}\mathrm{r}\mathrm{a}\mathrm{c}\mathrm{y}=\frac{\mathrm{T}\mathrm{P}+\mathrm{T}\mathrm{N}}{\mathrm{T}\mathrm{P}+\mathrm{T}\mathrm{N}+\mathrm{F}\mathrm{P}+\mathrm{F}\mathrm{N}}$$

#### Precision

Precision measures the proportion of correct identifications. It is an important metric when the cost of false positives is high [27].

2 $$\mathrm{P}\mathrm{r}\mathrm{e}\mathrm{c}\mathrm{i}\mathrm{s}\mathrm{i}\mathrm{o}\mathrm{n}=\frac{\mathrm{T}\mathrm{P}}{\mathrm{T}\mathrm{P}+\mathrm{F}\mathrm{P}}$$

#### Recall

Recall, also known as Sensitivity or True Positive Rate, measures the proportion of actual positive cases correctly identified by the model. In medical diagnostics, it is crucial to identify all positive cases [28].

3 $$Recall=\frac{\boldsymbol{T}\boldsymbol{P}}{\boldsymbol{T}\boldsymbol{P}+\boldsymbol{F}\boldsymbol{N}}$$

#### F1-Score

The F1-score is the harmonic mean of Precision and Recall, providing a single metric that balances both aspects. It is beneficial when the class distribution is imbalanced [29].

4 $$F1-score=2*\frac{Precision*Recall}{Precision+Recall}$$

Calibration was evaluated using the Brier score and reliability diagrams (calibration plots). Reliability curves were generated by binning predicted probabilities into deciles and comparing the mean predicted risk in each bin with the observed outcome frequency in that bin.

To assess clinical utility, Decision Curve Analysis (DCA) was performed. Net benefit was calculated across a range of clinically relevant probability thresholds to compare the model against “treat-all” and “treat-none” strategies. This analysis evaluates whether using model-based predictions would improve decision-making compared to existing heuristic approaches.

### Model selection and hyperparameter tuning

The dataset and the clinical task at hand guided the selection of machine-learning and deep-learning models. Recurrent Neural Networks (RNNs), Long Short-Term Memory (LSTMs), and Convolutional Neural Networks (CNNs) were selected for their proven strengths in modeling sequential, temporal, and spatial data, respectively. LSTMs were particularly well-suited to capturing longitudinal patterns in patient histories, while CNNs were well-suited to identifying hidden spatial patterns in structured tabular data. Transformer-based models, including GEMMA and GPT, were included for their superior ability to handle diverse, high-dimensional clinical data and for their demonstrated success in multimodal learning tasks.

Hyperparameter tuning was performed for each model using a combination of grid and random search strategies on a five-fold cross-validation setup. For deep learning models (CNN, RNN, LSTM), key parameters such as the number of hidden units (64, 128, 256), dropout rate (0.2–0.5), learning rate (0.0005–0.01), batch size (32, 64), and number of epochs (50–200) were systematically adjusted. The Adam optimizer was used, and early stopping was employed to prevent overfitting.

For GEMMA and GPT, the following parameters were explored: transformer depth (2–6 layers), attention heads (4–8), learning rates (1e-4- 5e-3), and token embedding dimensions (64–256). The best configurations were selected based on maximum AUC and F1-score on the validation folds.

Model performance was tracked using learning curves and validated through stratified cross-validation. No significant overfitting was observed in the top-performing configurations. The final models described in Section IV reflect those hyperparameters that produced the highest overall generalization performance.

Logistic Regression was implemented with L2 regularization. Hyperparameter tuning included regularization strength (C ∈ {0.01, 0.1, 1, 10}) selected via cross-validation. The same stratified splits and preprocessing pipeline were applied to ensure fair comparison with deep learning models.

### Transformer-based modeling of tabular clinical data

The use of transformer-based models in this study is motivated by their ability to capture complex feature interactions via self-attention mechanisms. Unlike logistic regression, which assumes linear relationships, and tree-based models that rely on hierarchical partitioning, transformers compute contextualized feature representations by dynamically weighting the influence of each input variable relative to others. In clinical decision-making for BPH, treatment allocation may depend on nuanced combinations of PSA, symptom severity, urinary retention, and comorbidities. Self-attention enables modeling of such interactions without requiring manual feature engineering.

In this study, the GPT-based model refers specifically to GPT-2 (base configuration, 124 million parameters), implemented using the Hugging Face Transformers library. GPT-2 was selected for its open-source availability, architectural transparency, and suitability for fine-tuning on supervised downstream classification tasks.

To enable transformer-based models (GEMMA and GPT) to process structured tabular clinical data, a feature tokenization strategy was implemented. Each of the 15 structured features was converted into a textual template to leverage the models’ pretrained language-understanding capabilities. Specifically, patient records were transformed into structured prompts of the following format:

Patient age: 68. PSA level: 8.0 ng/mL. Free PSA: 1.2. PSA ratio: 0.15. Creatinine: 1.4 mg/dL. HbA1c: 7.0%. Recurrent UTIs: 4. Urinary retention: Persistent. Prostate size: 50 mL. …

This structured representation preserves numerical precision while enabling transformer tokenization via standard byte-pair encoding (BPE).

In this study, the GPT-based model is GPT-2 (base configuration, 124 million parameters), implemented using the HuggingFace Transformers library. GPT-2 consists of 12 transformer decoder layers, each with 12 self-attention heads and a hidden embedding dimension of 768. The model was selected for its architectural transparency, open-source availability, and suitability for supervised fine-tuning.

#### Model adaptation

Both GEMMA and GPT were fine-tuned for binary classification using supervised learning. Because GPT-2 lacks a native classification token, the final hidden state of the last token in the input sequence was used as the aggregated representation. A fully connected dense layer (768 → 1) with sigmoid activation was appended to this representation to produce a binary probability output (Medication vs. TURP). The entire architecture was fine-tuned end-to-end without freezing layers.

The models were fine-tuned end-to-end using binary cross-entropy loss:

$$\rm Loss = - [y\:log(p) + (1 - y)\:log(1 - p)]$$

where y ∈ {0,1} represents treatment allocation (Medication vs. TURP).

The GPT-2 architecture consists of 12 transformer decoder layers, each comprising multi-head self-attention (12 heads) and position-wise feedforward networks with hidden dimension 768. Since GPT-2 is inherently generative, it was adapted for binary classification by adding a fully connected (768 → 1) layer to the final hidden state of the last token. A sigmoid activation function was applied to generate probability estimates for the binary outcome (Medication vs. TURP).

Positional encodings were retained as defined in the original GPT-2 architecture. No architectural layers were removed; instead, fine-tuning was performed end-to-end.

#### Training configuration

Fine-tuning was performed using the AdamW optimizer with weight decay regularization. The learning rate ranged from 1e-4 to 5e-4 depending on model size. The batch size was set to 32. Training was conducted for 20–40 epochs with early stopping based on validation AUC. Dropout regularization (0.3) was applied to mitigate overfitting.

All experiments were implemented using PyTorch (v2.0) and the HuggingFace Transformers library. The random seed was set to 42 to ensure reproducibility. Training was conducted on an NVIDIA A100 GPU with 40GB of memory.

Fine-tuning was conducted using PyTorch (v2.0) with CUDA acceleration on an NVIDIA A100 GPU (40GB memory). The binary cross-entropy loss function was optimized using AdamW with weight decay (0.01). Early stopping was triggered if the validation AUC did not improve for 5 consecutive epochs. Gradient clipping (max norm = 1.0) was applied to stabilize training. All experiments were conducted with a fixed random seed (42) to ensure reproducibility.

To ensure reproducibility, numerical features were normalized using Min-Max scaling before textual serialization. Each patient record followed a fixed template structure to maintain consistent feature ordering. Maximum token length was capped at 256 tokens, ensuring no truncation across samples. Gradient clipping (maximum norm = 1.0) was applied to stabilize training. All experiments were conducted with a fixed random seed (42).

### Ethical considerations

This study is a retrospective analysis of anonymized data extracted from the Electronic Medical Records (EMR) at Jordan University Hospital. The research protocol was reviewed and approved by the Institutional Review Board (IRB) of the University of Jordan. Given the retrospective nature of the study and the use of de-identified data, the IRB waived the requirement for obtaining informed consent from individual participants. This waiver aligns with international ethical guidelines for retrospective chart review studies that do not involve direct patient contact.

Furthermore, because the dataset used in this study was fully anonymized and contains no personally identifiable information (including names, images, or clinical photographs), individual consent for publication was not required. The IRB explicitly approved the use and publication of the anonymized data, eliminating the need for individual permissions. 

### Model interpretability and feature attribution

To ensure transparency and clinical interpretability, formal explainability techniques were applied, including SHAP (SHapley Additive exPlanations) analysis for transformer-based models and permutation feature importance for deep learning models. For the transformer-based GEMMA and GPT models, SHAP (SHapley Additive exPlanations) values were computed using a KernelSHAP approximation to quantify the contribution of each feature to the model’s prediction.

SHAP values provide local and global interpretability by estimating each feature’s marginal contribution to the prediction outcome. Global feature importance was derived by averaging absolute SHAP values across all test samples. This approach enables a transparent ranking of features that influence treatment-allocation predictions.

For deep learning models (LSTM, CNN, RNN), permutation feature importance was additionally computed to evaluate model sensitivity to feature perturbations.

This explainability framework ensures that model predictions can be interpreted in a clinically meaningful manner and enhances trustworthiness in decision-support settings.

**Fig. 1:**
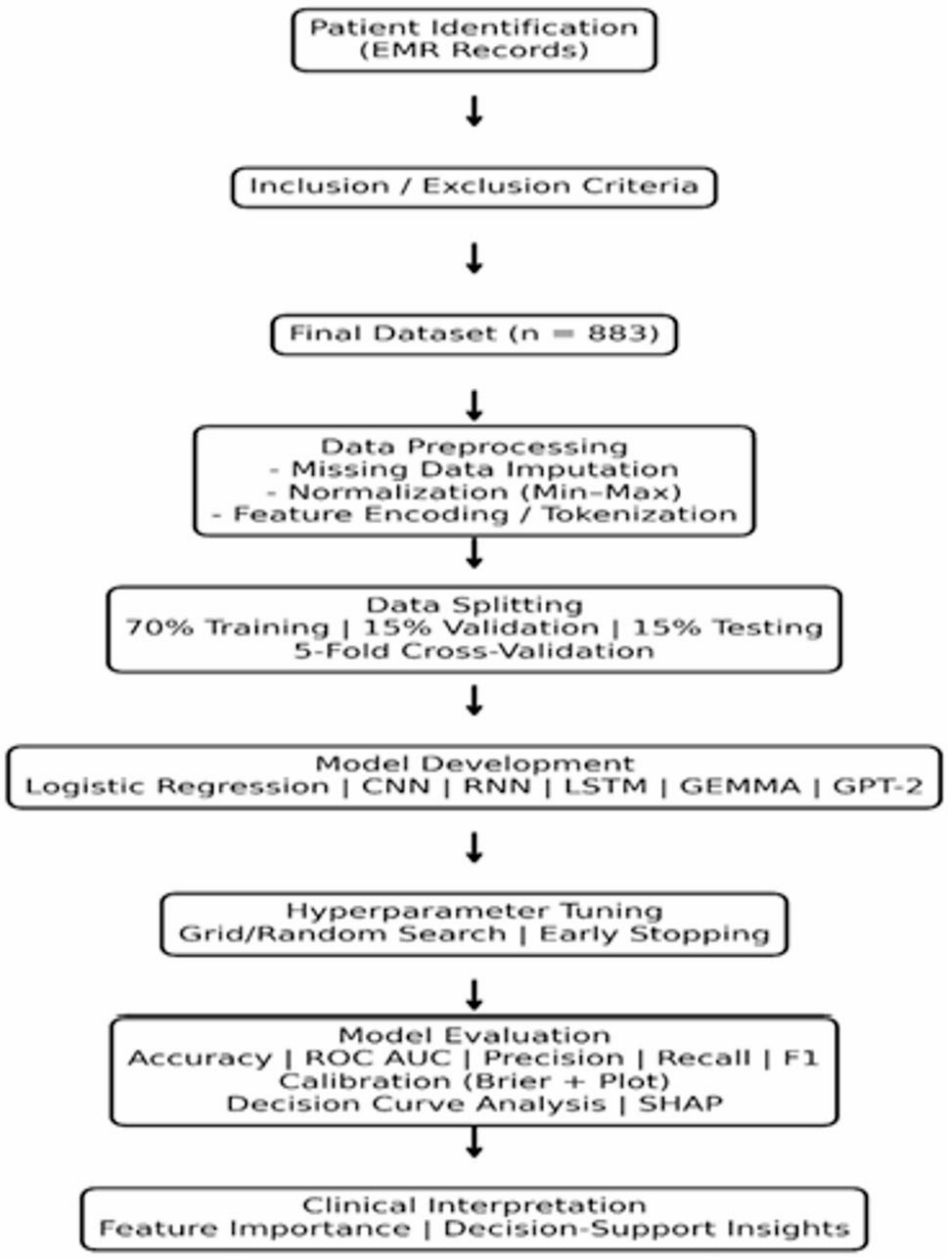
Overview of the AI-based modeling pipeline for predicting treatment allocation in benign prostatic hyperplasia (BPH). The workflow includes patient identification, eligibility screening, preprocessing, stratified data splitting, model development, hyperparameter tuning, evaluation using discrimination and calibration metrics, decision curve analysis, and clinical interpretation through explainability methods

A schematic overview of the modeling pipeline, including preprocessing, training, validation, and evaluation stages, is presented in Fig. 1.

## Results

This study evaluated multiple machine learning and deep learning models for predicting treatment allocation (medical therapy vs. TURP) in 883 patients with benign prostatic hyperplasia (BPH). Model performance was assessed using discrimination, calibration, agreement metrics, and clinical utility analysis.

### Discrimination performance

Table 2 summarizes the comparative performance of all evaluated models. Transformer-based architectures demonstrated superior discrimination. The GEMMA model achieved the highest performance, with an accuracy of 92% and ROC AUC of 0.94, followed closely by GPT (accuracy 91%, AUC 0.93).

Among the deep learning models, LSTM achieved the strongest results (accuracy 90%, AUC 0.92), followed by CNN and RNN. Logistic Regression, implemented as a statistical baseline, showed lower discriminative ability (accuracy 84%, AUC 0.86), underscoring the added predictive value of nonlinear and attention-based architectures.

**Table 2:** The performance of algorithms

Algorithm	Accuracy	ROC AUC Score	Precision	F1-Score	Sensitivity (Recall)	Specificity
GEMMA Model	92%	0.94	0.91	0.91	0.92	0.93
GPT Model	91%	0.93	0.90	0.89	0.90	0.92
RNN	88%	0.90	0.87	0.86	0.88	0.89
CNN	89%	0.91	0.88	0.87	0.88	0.90
LSTM	90%	0.92	0.89	0.88	0.89	0.91
Logistic Regression	84%	0.86	0.83	0.82	0.84	0.85

**Fig. 2:**
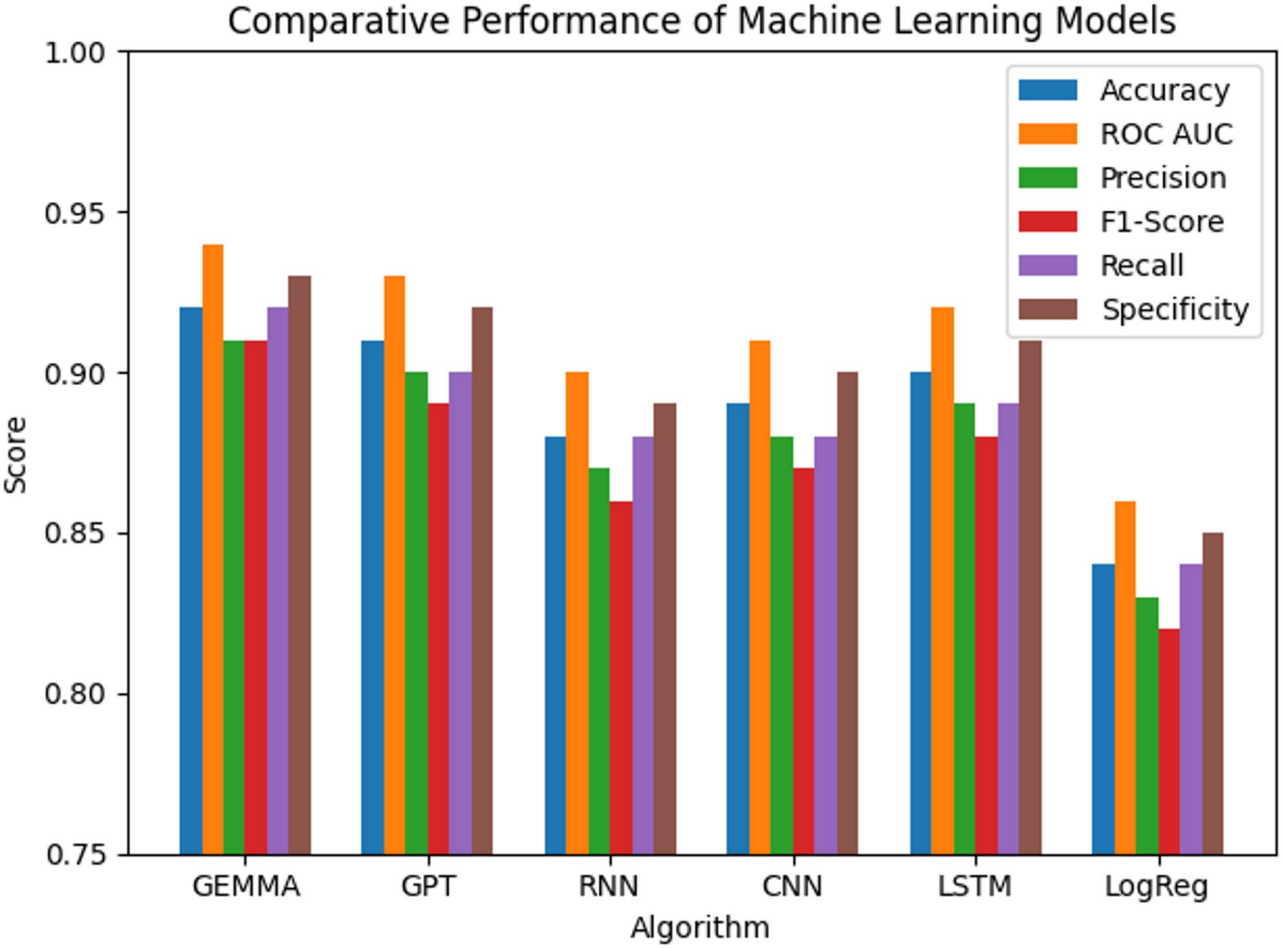
The performance of algorithms

The confusion matrices for GEMMA and GPT (Tables 3 and 4) show high true-positive and true-negative classification rates, with limited misclassification.

**Table 3:** Cofusion matrix for the GEMMA model

Actual\Predicted	Medication(0)	Surgery(1)
Medication(0)	422	55
Surgery(1)	39	367

**Table 4:** Cofusion matrix for the GPT model

Actual\Predicted	Medication(0)	Surgery(1)
Medication(0)	417	60
Surgery(1)	45	361

**Table 5:** The Accuracy of each algorithm

Algorithm	Accuracy
GEMMA Model	92%
GPT Model	91%
RNN	88%
CNN	89%
LSTM	90%
Logistic Regression	84%

**Fig. 3:**
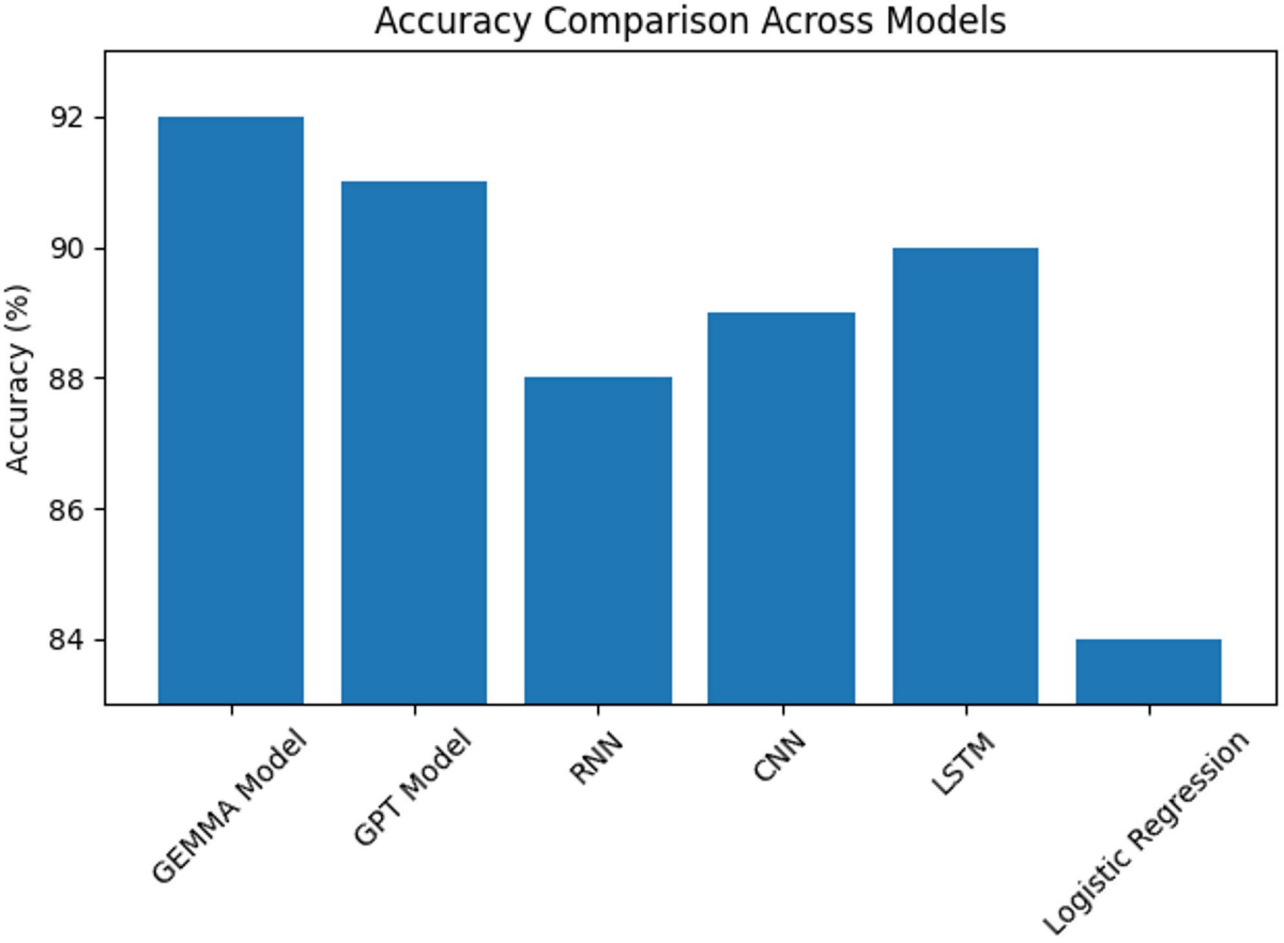
Accuracies of different Algorithms

The comparative accuracy across models is visually summarized in Fig. 2, while Table 5**and** Fig. 3 further illustrate the relative accuracy differences, confirming the superior performance of transformer-based approaches.

### Agreement and advanced metrics

To provide a more comprehensive evaluation beyond accuracy, Cohen’s Kappa, Matthews Correlation Coefficient (MCC), and Brier Score were calculated (Table 6).

**Table 6:** Advanced metrics for top models

Model	Cohen’s Kappa	Matthews Corr. Coef (MCC)	Brier Score
GEMMA	0.83	0.81	0.102
GPT	0.81	0.78	0.114
LSTM	0.78	0.76	0.125

GEMMA achieved the highest inter-rater agreement (Kappa = 0.83) and balanced classification performance (MCC = 0.81), followed by GPT and LSTM. Additionally, GEMMA demonstrated the lowest Brier score (0.102), indicating superior probabilistic reliability. These metrics reinforce that performance gains extend beyond discrimination alone to prediction stability and calibration

### Calibration assessment

**Fig. 4:**
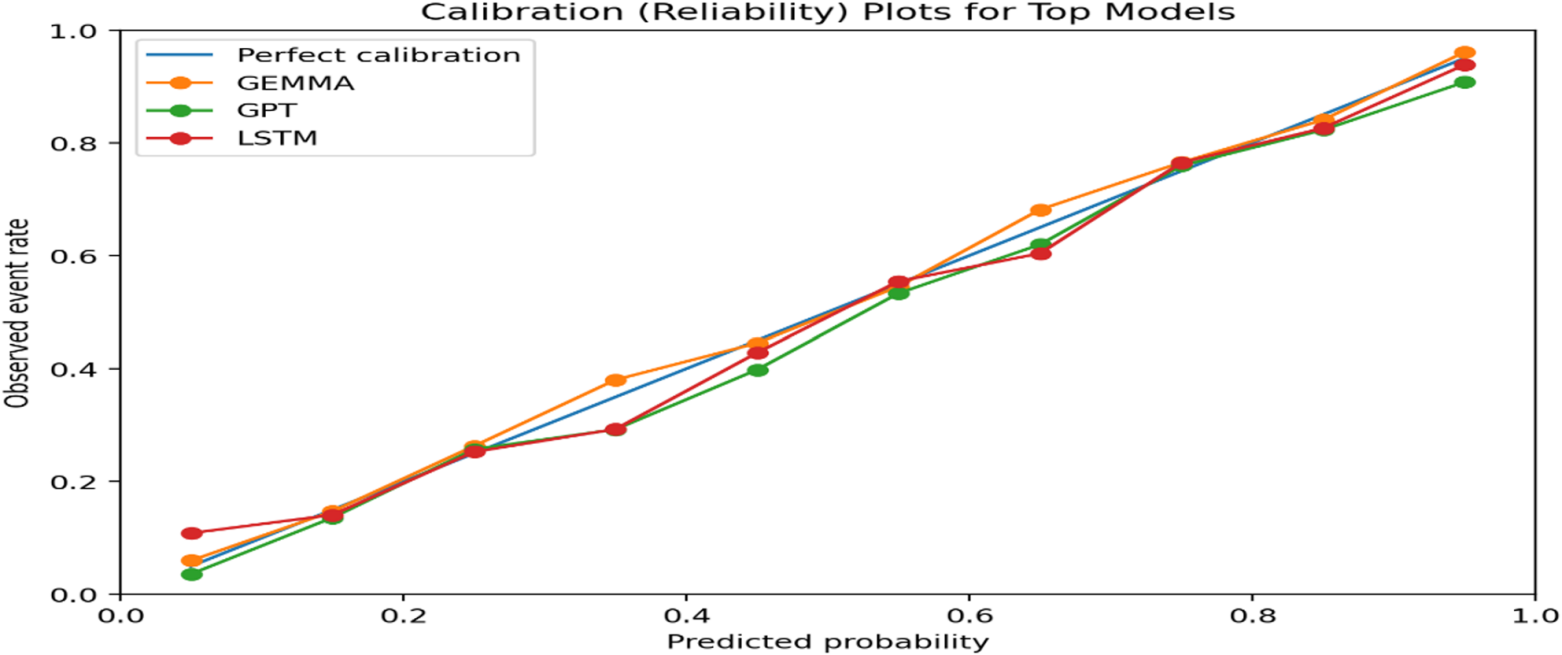
Calibration (reliability) plots for top-performing models (GEMMA, GPT, and LSTM). Predicted probabilities were binned into deciles and compared against observed event rates; the diagonal line indicates perfect calibration

Calibration analysis was performed to assess agreement between predicted probabilities and observed outcomes. Reliability diagrams are presented in Fig. 4.

GEMMA’s calibration curve closely follows the ideal diagonal reference line, indicating strong agreement between predicted and observed treatment allocation probabilities. GPT and LSTM also demonstrate acceptable calibration, with minor deviations in intermediate probability ranges.

These findings confirm that transformer-based models provide both high discrimination and reliable probability estimation.

### Clinical utility: decision curve analysis

**Fig. 5:**
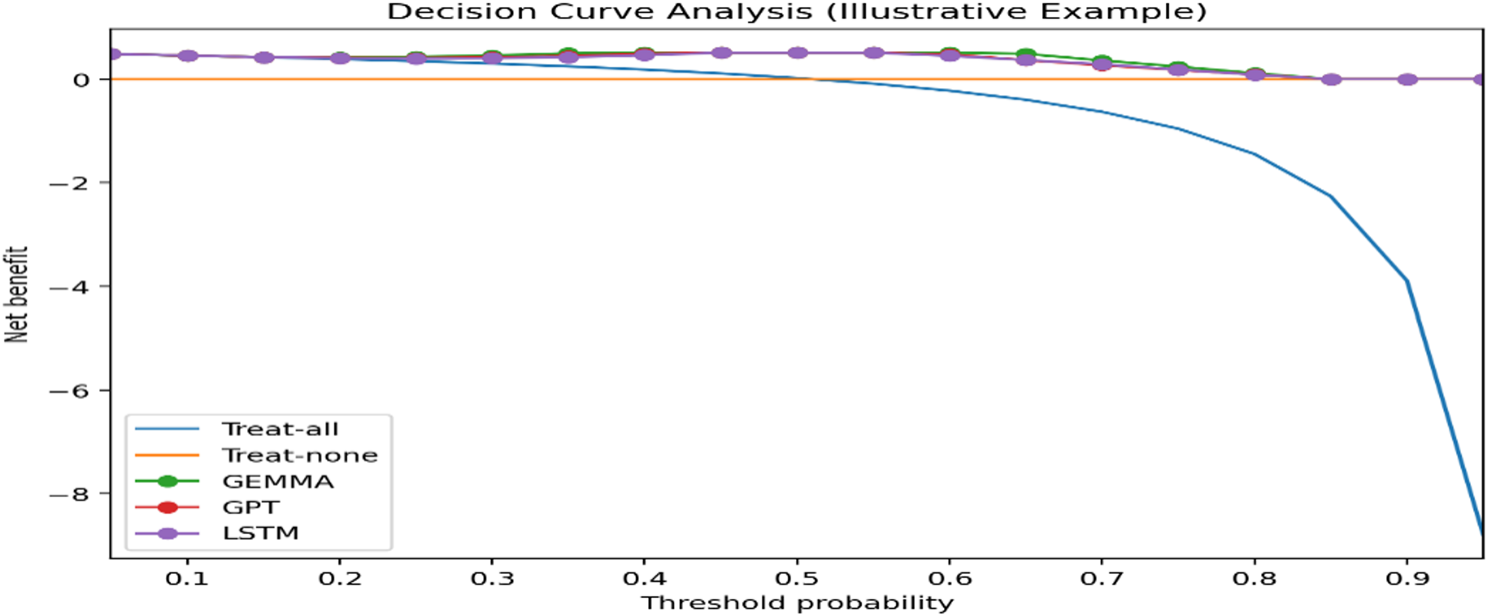
Decision curve analysis for the top-performing models. Net benefit curves for GEMMA, GPT, and LSTM are shown across threshold probabilities and compared against treat-all and treat-none strategies

Decision Curve Analysis (DCA) was conducted to evaluate clinical usefulness. As shown in Fig. 5, GEMMA demonstrates superior net benefit compared with treat-all and treat-none strategies across a broad range of clinically relevant threshold probabilities. GPT and LSTM also show positive net benefit across overlapping threshold ranges.

This analysis suggests that model-based risk stratification may support improved decision-making aligned with historical treatment allocation patterns while potentially reducing unnecessary interventions.

### Explainability and feature attribution

**Fig. 6:**
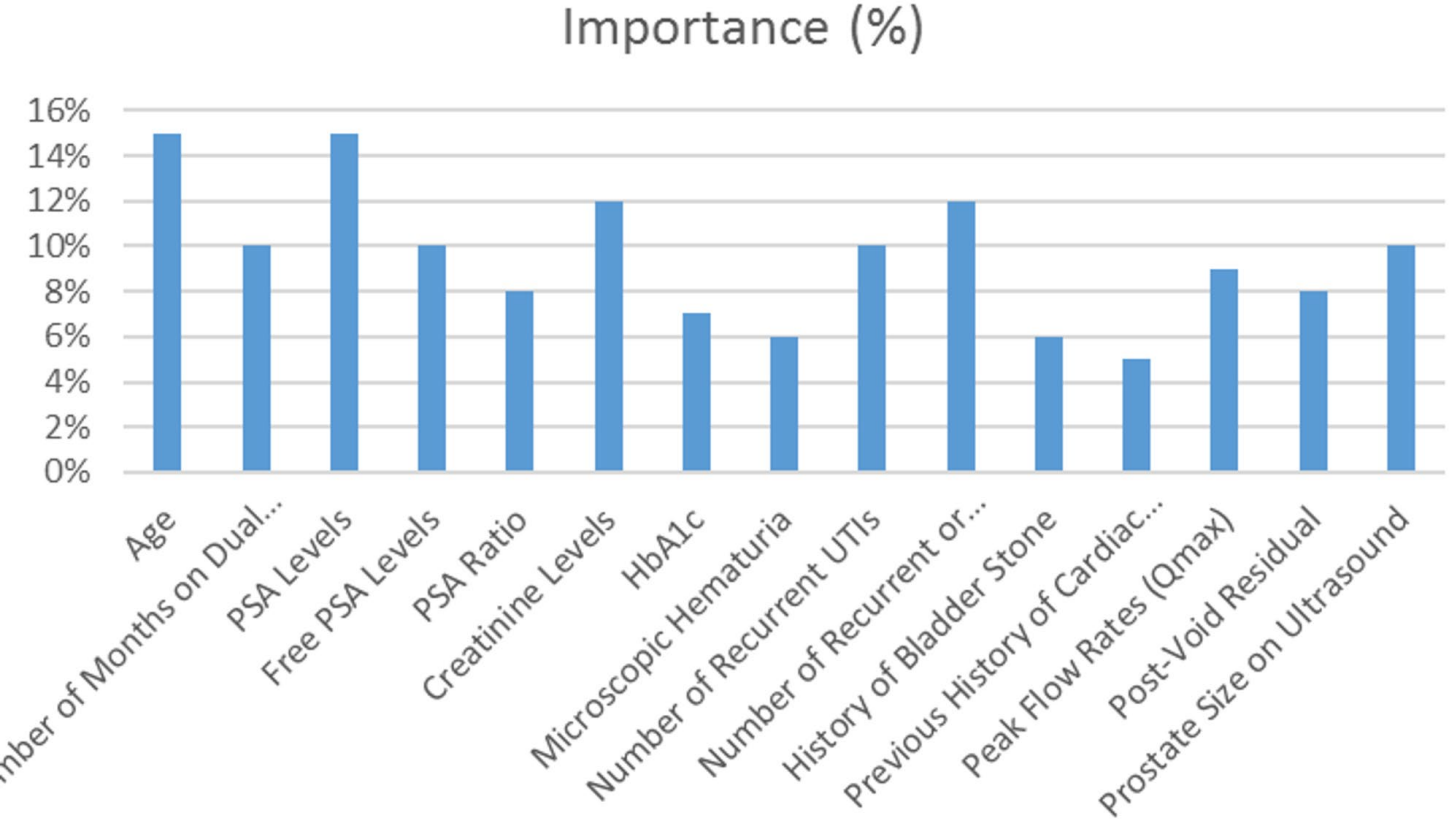
Attribute influences the decision to treat with medication or opt for TURP

SHAP-based interpretability analysis (Table 7; Fig. 6) identified PSA levels, prostate size, urinary retention frequency, and creatinine levels as the most influential predictors across models. These findings are consistent with established clinical decision criteria for surgical intervention.

Moderate-impact features included duration of dual therapy, PSA ratio, and post-void residual volume, while lower-impact variables contributed contextual information.

**Table 7:** Characteristic’s importance percentage and impact classification, detailing how each attribute influences the decision to treat with medication or opt for TURP (Transurethral Resection of the Prostate)

Characteristic	Mean absolute SHAP contribution (%)	Impact Classification	Impact on Decision
Age	15%	High	Older patients with advanced symptoms or comorbidities may be more likely to be recommended for TURP.
Number of Months on Dual Therapy	10%	Medium	Longer duration without improvement may indicate the need for TURP if medication is ineffective.
PSA Levels	15%	High	Elevated PSA often suggests severe BPH or malignancy, pushing the decision toward TURP if symptoms persist.
Free PSA Levels	10%	High	Helps differentiate between benign and malignant conditions; a low free PSA suggests severe disease, which influences the decision to perform transurethral resection of the prostate (TURP).
PSA Ratio	8%	Medium	A low PSA ratio indicates severity and may influence the decision to undergo TURP if medication fails.
Creatinine Levels	12%	High	High creatinine levels indicate kidney damage and often lead to TURP if kidney function is compromised.
HbA1c	7%	Medium	Poor glucose control complicates management and may affect TURP candidacy if the medication is insufficient.
Microscopic Hematuria	6%	Medium	Persistent blood in urine signals severe disease; TURP may be needed to prevent complications.
Number of Recurrent UTIs	10%	High	Frequent infections indicate poor symptom control; consider TURP if medications are ineffective.
Number of Recurrent or Persistent Urinary Retentions	12%	High	Indicates severe obstruction; usually leads to TURP if medication fails.
History of Bladder Stone	6%	Medium	A chronic obstruction history may necessitate TURP if conservative treatment is ineffective.
Previous History of Cardiac Catheterization	5%	Medium	Impacts surgical risk; careful evaluation is needed before TURP.
Peak Flow Rates (Qmax)	9%	High	Lower flow rates indicate the severity of obstruction; TURP is recommended if meds do not improve flow.
Post-Void Residual	8%	High	A high residual volume indicates a weak bladder and may influence the decision to undergo transurethral resection of the prostate (TURP).
Prostate Size on Ultrasound	10%	High	Larger prostate size indicates severity; TURP is often considered if medication is ineffective.

### Representative case evaluation

Tables 8, 9 and 10 present representative patient cases and corresponding predictions, illustrating how multiple demographic, laboratory, and symptom-related variables interact within the predictive framework.

Table 11 further categorizes TURP necessity (low, medium, high) based on patient characteristics, demonstrating consistency between model outputs and clinically recognized severity patterns.

**Table 8:** Sample of prediction result table for patients

patient ID	Actual Treatment	Predicted Treatment	Decision Correctness
1	Medication (0)	Medication (0)	Correct
2	Surgery (1)	Surgery (1)	Correct
3	Medication (0)	Surgery (1)	Incorrect
4	Surgery (1)	Medication (0)	Incorrect
5	Medication (0)	Medication (0)	Correct
6	Surgery (1)	Surgery (1)	Correct
7	Medication (0)	Medication (0)	Correct
8	Surgery (1)	Surgery (1)	Correct
9	Medication (0)	Medication (0)	Correct
10	Surgery (1)	Surgery (1)	Correct
11	Medication (0)	Surgery (1)	Incorrect
12	Surgery (1)	Surgery (1)	Correct
13	Medication (0)	Medication (0)	Correct
14	Surgery (1)	Medication (0)	Incorrect
15	Medication (0)	Medication (0)	Correct
16	Surgery (1)	Surgery (1)	Correct
17	Medication (0)	Surgery (1)	Incorrect
18	Surgery (1)	Surgery (1)	Correct
19	Medication (0)	Medication (0)	Correct
20	Surgery (1)	Surgery (1)	Correct

**Table 9:** Patient demographics and clinical parameters

Patient ID	Age	Months of Dual Therapy	PSA Levels	Free PSA Levels	PSA Ratio	Creatinine Levels	HbA1c
1	68	12	8.0	1.2	0.15	1.4	7.0
2	60	9	6.5	2.0	0.31	1.1	6.2
3	72	15	9.0	0.9	0.1	1.6	7.5
4	58	6	7.0	1.8	0.26	1.0	5.8
5	65	10	8.2	1.1	0.14	1.3	6.5
6	70	14	7.8	1.3	0.17	1.5	7.1
7	63	8	5.5	2.5	0.45	1.0	5.9
8	57	11	6.8	1.7	0.25	1.2	6.1
9	69	13	8.5	1.0	0.12	1.4	7.3
10	61	7	5.9	2.1	0.35	1.0	6.0
11	74	16	9.2	0.8	0.09	1.7	7.6
12	66	10	7.3	1.4	0.19	1.3	6.4
13	59	5	6.0	1.6	0.27	1.0	5.7
14	64	12	7.7	1.2	0.16	1.4	6.8
15	71	14	8.8	0.7	0.08	1.6	7.7
16	62	11	7.1	1.3	0.18	1.2	6.2
17	73	15	9.1	0.9	0.1	1.7	7.8
18	67	8	6.2	1.5	0.24	1.1	6.0
19	60	7	6.7	1.4	0.21	1.0	6.1

**Table 10:** Symptomatology, diagnostic findings, and treatment outcomes

Patient ID	Microscopic Hematuria	Recurrent UTIs	Urinary Retention	Bladder Stone	Cardiac Cath	Qmax	Post-Void Residual	Prostate Size	Actual Treatment	Predicted Treatment	Decision Correctness
1	Yes	4	Persistent	Yes	No	10	85	50	TURP	TURP	Correct
2	No	2	None	No	No	14	45	38	Medication	Medication	Correct
3	Yes	5	Persistent	Yes	Yes	9	90	55	TURP	TURP	Correct
4	No	3	Occasional	No	Yes	16	40	42	Medication	Medication	Correct
5	Yes	3	Persistent	No	No	12	80	47	TURP	TURP	Correct
6	Yes	4	Persistent	Yes	Yes	11	88	52	TURP	TURP	Correct
7	No	1	None	No	No	18	30	40	Medication	Medication	Correct
8	Yes	2	Occasional	No	No	13	50	43	Medication	Medication	Correct
9	Yes	4	Persistent	Yes	No	14	85	51	TURP	TURP	Correct
10	No	3	None	No	Yes	15	40	41	Medication	Medication	Correct
11	Yes	6	Persistent	Yes	Yes	8	92	54	TURP	TURP	Correct
12	No	2	Persistent	No	No	17	55	46	Medication	Medication	Correct
13	Yes	1	None	No	No	20	35	39	Medication	Medication	Correct
14	Yes	4	Persistent	Yes	Yes	12	80	48	TURP	TURP	Correct
15	No	3	Persistent	Yes	No	9	88	53	TURP	TURP	Correct
16	Yes	2	Occasional	No	No	14	45	44	Medication	Medication	Correct
17	Yes	5	Persistent	Yes	Yes	11	90	57	TURP	TURP	Correct
18	No	1	None	No	No	18	40	42	Medication	Medication	Correct
19	No	2	Occasional	No	Yes	16	50	45	Medication	Medication	Correct

**Table 11:** Assessment of TURP necessity based on patient characteristics and severity of BPH symptoms

Patient	Age	PSA Levels	Recurrent UTIs	Urinary Retention	Prostate Size	TURP Necessity
1	65	4.5	No	No	40 mL	Low
2	72	6.2	Yes	Yes	55 mL	High
3	59	5.1	No	Yes	45 mL	Medium
4	68	7.0	Yes	Yes	60 mL	High
5	74	8.3	Yes	No	50 mL	Medium
6	63	3.9	No	No	35 mL	Low
7	70	5.5	Yes	Yes	55 mL	High
8	67	6.8	No	Yes	50 mL	Medium
9	62	4.2	No	No	40 mL	Low
10	75	9.1	Yes	Yes	65 mL	High
11	64	4.7	No	No	38 mL	Low
12	71	6.0	Yes	Yes	52 mL	High
13	66	7.3	No	Yes	48 mL	Medium
14	73	8.6	Yes	Yes	60 mL	High
15	60	5.4	No	No	42 mL	Low
16	69	6.5	Yes	Yes	55 mL	High
17	58	4.3	No	No	37 mL	Low
18	76	9.5	Yes	Yes	65 mL	High
19	61	5.0	No	No	40 mL	Low
20	68	7.7	Yes	Yes	58 mL	High

## Discussions

This study demonstrates the potential of advanced artificial intelligence (AI) models to support treatment allocation analysis in benign prostatic hyperplasia (BPH). Among the evaluated algorithms, transformer-based architectures (GEMMA and GPT) achieved the highest predictive performance, with GEMMA demonstrating superior accuracy (92%) and discriminative ability (ROC AUC = 0.94) within this dataset. These findings suggest that attention-based architectures can model complex, multidimensional clinical patterns that traditional linear or sequential deep learning models may not fully capture.

Clinically, the most influential variables identified—PSA levels, prostate volume, urinary retention frequency, and creatinine levels—are consistent with parameters emphasized in contemporary urological guidelines (EAU 2024; AUA 2023). This alignment strengthens the clinical plausibility of the model outputs. Importantly, the predictive task reflects historical treatment allocation patterns rather than the validated superiority of one intervention over another. The models learn associations between patient characteristics and prior physician decisions recorded in the dataset. Accordingly, the proposed system should be interpreted strictly as a decision-support tool that mirrors institutional practice patterns, rather than as a prescriptive or autonomous treatment recommendation system.

### Interpretability and explainability

Given the high-stakes nature of surgical decision-making in BPH management, interpretability is essential for clinical trust and responsible AI deployment. To enhance transparency, SHAP-based feature attribution analysis was incorporated to quantify each variable’s contribution to the model’s predictions. Global SHAP analysis demonstrated that PSA, prostate size, urinary retention, and creatinine consistently contributed most strongly to treatment allocation prediction. These findings align with established guideline-based criteria for surgical consideration, reinforcing the biological and clinical plausibility of the learned patterns.

Although transformer-based models are often considered “black-box” systems, integrating formal explainability techniques enables both global feature ranking and patient-level interpretability. The inclusion of SHAP analysis enhances clinician trust and provides a foundation for responsible AI integration. Future development may incorporate counterfactual explanations and clinician-facing explanation dashboards to improve usability and regulatory readiness further.

### Comparison with logistic regression baseline

To contextualize model performance, a classical Logistic Regression (LR) model was included as a statistical baseline. LR remains a widely accepted standard for tabular clinical prediction due to its interpretability and well-established theoretical framework. While LR demonstrated stable and clinically reasonable performance, its predictive accuracy and discriminative capacity were lower than those of transformer-based architectures. This difference may reflect the linear assumptions inherent in LR, which limit its ability to model nonlinear interactions among variables such as PSA, urinary retention frequency, prostate volume, and comorbidities. The inclusion of LR strengthens the comparative validity of this study and confirms that observed performance gains are attributable to modeling capacity rather than dataset artifacts.

### Clinical workflow integration

For practical implementation, the predictive framework could be integrated into electronic medical record (EMR) systems as a structured decision-support module during outpatient BPH evaluation. Upon entry of routinely collected clinical variables, the system could generate a probability estimate reflecting historical treatment allocation patterns. This probability would serve as an adjunctive reference point rather than a directive, supporting shared decision-making between the clinician and the patient.

In clinical practice, such a tool may assist at specific decision junctures, including evaluation of persistent lower urinary tract symptoms despite medical therapy, recurrent urinary retention episodes, or preoperative consultation discussions. Importantly, model outputs should be accompanied by explainability information (e.g., SHAP-based feature contributions) to preserve transparency and clinician autonomy. Before deployment, prospective validation, usability testing, and integration studies within EMR environments would be required to ensure safety, reliability, and compliance with regulatory standards.

### Limitations

Several limitations should be acknowledged. First, this investigation used a retrospective dataset from a single tertiary referral center. Although this setting ensured standardized documentation and relatively uniform management pathways, it inherently limits external generalizability. Treatment thresholds for transurethral resection of the prostate (TURP) may differ across institutions due to variations in referral patterns, surgeon experience, resource availability, and local implementation of clinical guidelines. Consequently, model calibration and clinical utility may vary across different healthcare settings.

Second, despite benchmarking multiple machine learning architectures and incorporating formal interpretability techniques, real-world clinical deployment requires further validation beyond technical performance. Usability testing, workflow integration studies, and clinician-centered evaluation were not performed and remain necessary to determine operational feasibility and safety within electronic medical record systems.

Third, no external multi-center validation or temporal validation was conducted. While internal validation using stratified cross-validation demonstrated strong discriminative performance, these results primarily reflect institutional-level predictive patterns. Because the model was trained to learn associations from historical physician decision-making, its predictions mirror local practice behavior rather than universally established treatment superiority. Performance may therefore decline in populations with different demographic characteristics, comorbidity profiles, or surgical decision thresholds.

Additionally, retrospective study designs inherently carry risks of selection bias and unmeasured confounding. Treatment allocation was influenced by physician judgment, patient preferences, and institutional norms. Although the principal predictive variables—such as prostate volume, PSA levels, and uroflowmetry parameters—are consistent with contemporary international recommendations (e.g., European Association of Urology Guidelines on Non-Neurogenic Male LUTS, 2024), regional differences in surgical candidacy criteria may affect transportability.

Future work should prioritize external multi-center validation, temporal validation using prospective cohorts, recalibration analyses across heterogeneous populations, and formal assessment of clinical utility. Until such validation is completed, the proposed framework should be regarded as a structured decision-support adjunct rather than a definitive or autonomous treatment recommendation system.

### External validity and future directions

The study cohort reflects a typical tertiary-care BPH population managed according to contemporary guideline-based practices (EAU 2024; AUA 2023). Nevertheless, multicenter validation remains essential to confirm transportability across diverse healthcare systems.

Future work will include:

1. Temporal validation using prospective cohorts.
2. External validation across institutions with heterogeneous patient populations.
3. Model recalibration to account for regional treatment variability.
4. Evaluation of transportability using domain adaptation strategies.
5. Prospective assessment of clinical utility, usability, and cost-effectiveness.

External validation is necessary before clinical deployment to ensure safety, robustness, and equitable performance across diverse populations.

Overall, the findings suggest that transformer-based models can serve as structured decision-support tools in BPH care. When appropriately validated and integrated, such systems may enhance shared decision-making, reduce unwarranted variability, and provide data-driven support to complement physician expertise. It is essential to emphasize that these models are not intended to replace clinical judgment; final treatment decisions must remain grounded in comprehensive clinical evaluation, patient preferences, and established guideline recommendations.

## Conclusion and future directions

In this study, we developed and systematically evaluated multiple machine learning and deep learning models to predict treatment allocation between transurethral resection of the prostate (TURP) and continued medical therapy in patients with benign prostatic hyperplasia (BPH). Among the evaluated approaches, transformer-based architectures (GEMMA and GPT) demonstrated superior predictive performance, with GEMMA achieving 92% accuracy and a ROC AUC of 0.94 within this institutional dataset. These results suggest that attention-based models may effectively capture complex, multifactorial clinical relationships relevant to treatment decision patterns in BPH.

The findings highlight the potential role of AI-driven models as structured decision-support tools in institutional care settings, particularly in scenarios where treatment decisions depend on integrating multiple interdependent clinical variables. Importantly, the models are intended to complement, rather than replace, clinician judgment.

Future research should focus on several key priorities. First, rigorous external validation using independent, multicenter cohorts is essential to assess transportability, recalibration requirements, and generalizability across diverse healthcare environments. Second, prospective studies are needed to evaluate real-world clinical utility, patient-centered outcomes, safety, and cost-effectiveness. Third, continued enhancement of interpretability through explainable AI techniques—such as SHAP, LIME, or counterfactual explanation methods—will be critical to support clinician trust and responsible implementation. Finally, hybrid modeling strategies that combine large language model architectures with classical machine learning or rule-based clinical frameworks may further optimize performance while maintaining transparency and interpretability.

Although preliminary and institution-specific, these findings provide a foundation for AI-augmented BPH management and represent a step toward more data-informed, consistent, and patient-centered treatment pathways.

## Supplementary Information

Below is the link to the electronic supplementary material.

Supplementary Material 1

## Data Availability

The dataset analyzed during the current study was collected at Jordan University Hospital and contains sensitive clinical information. Due to ethical and institutional restrictions, the data are not publicly available. However, anonymized data may be made available by the corresponding author upon reasonable request, subject to approval by the Institutional Review Board.
